# Myopia, corneal endothelial cell density and morphology in a Japanese population-based cross-sectional study: the JPHC-NEXT Eye Study

**DOI:** 10.1038/s41598-021-85617-4

**Published:** 2021-03-18

**Authors:** Naohiko Aketa, Miki Uchino, Motoko Kawashima, Yuichi Uchino, Kenya Yuki, Yoko Ozawa, Mariko Sasaki, Kazumasa Yamagishi, Norie Sawada, Shoichiro Tsugane, Kazuo Tsubota, Hiroyasu Iso

**Affiliations:** 1 Department of Ophthalmology, Keio University School of Medicine, 35 Shinanomachi, Shinjuku-ku, Tokyo 160-8582 Japan; 2 Department of Public Health Medicine, Faculty of Medicine, and Health Services Research and Development Center, University of Tsukuba, Tsukuba, Japan; 3 Ibaraki Western Medical Center, Chikusei, Japan; 4 Epidemiology and Prevention Group, Center for Public Health Sciences, National Cancer Center, Tokyo, Japan; 5 Tsubota Laboratory, Inc., Tokyo, Japan; 6 Department of Public Health, Graduate School of Medicine, Osaka University, Suita, Japan

**Keywords:** Eye diseases, Corneal diseases

## Abstract

This population-based cross-sectional study was performed to determine the mean corneal endothelial cell density (ECD), coefficient of variation (CV), and hexagonality (HEX), and their associations with myopia in Japanese adults living in Chikusei city. Of 7109 participants with available data, 5713 (2331 male and 3382 female) participants were eligible for analysis. After assessing the relationship between participant characteristics and spherical equivalent refraction (SER), the association of SER with the abnormal value of ECD (< 2000 cells/mm), CV (≥ 0.40), and HEX (≤ 50%) were determined using the logistic regression models adjusting for potential confounders (age, intraocular pressure, keratometric power, height, and antihypertensive drug use). In male participants, there was no statistically significant relationships between SER and endothelial parameters. In female participants, compared to emmetropia, SER ≤ − 6 D had significantly higher odds ratio (OR) of having the abnormal value of CV (OR = 2.07, 95% confidence interval [CI] 1.39–3.10) and HEX (OR = 2.04, 95% CI 1.29–3.23), adjusted for potential confounders, indicating that the high myopia was associated with the abnormal values of CV and HEX. Further adjustment for contact lenses wear partly attenuated these associations. Association between the SER and ECD was not detected.

## Introduction

Corneal endothelial cells play an important role in maintaining corneal clarity. Corneal endothelial cell density (ECD) loss, which is caused by various reasons such as intraocular surgery^1–4^, trauma^5^, inflammation^6,7^, and the aging process^8–23^, can lead to visual impairment in severe cases. Hence, it is clinically important to protect the corneal endothelial cells to prevent from potential visual deterioration. A recent study has shown that severe iris damage might be related to ECD loss after keratoplasty^7^ via aqueous cytokine interaction^24^. Although new insights about ECD have been reported lately, epidemiological evidence on ECD are still to be clarified.

The normal range of mean ECD varies depending upon race and sex^10,16–23^. Previous studies have examined ECD and its morphological characteristics, and most of them showed the ECD decrease is related to the increase in the variation of individual cell areas, i.e. coefficient of variation (CV), and decrease in the number of hexagonally-shaped endothelial cells, i.e. hexagonality (HEX). Some studies reported that high myopia was an independent factor that is associated with low ECD and abnormal endothelial morphology^25–27^.

Nowadays, alarming increase of myopia is noted worldwide, especially in teenagers and young adults^28,29^. Ocular conditions associated with myopia may cause irreversible vision loss^30^, and is already becoming a leading cause of blindness in many countries^31^. Some environmental factors such as reduced outdoor activities have found to be associated with myopia progression in young people, and several genes are known to be associated with high myopia^32^. Considering this myopia boom, it is now important to clarify the epidemiological relationships between myopia and ECD, CV, and HEX.

Based on the former findings, we hypothesized that myopia affect ECD and endothelial morphology, and examined the relationships between spherical equivalent refraction (SER) and prevalence of abnormal value of ECD, CV, and HEX in Japanese general population. To our knowledge, this is the largest epidemiological study providing robust data on normal ECD, its morphology and associated factors in the world.

## Results

### Participant demographics and inter-sex differences of ECD, CV and HEX

Among 5713 eligible participants, 2331 were male (mean age ± standard deviation: 64.2 ± 10.3 years) and 3382 were female (mean age: 61.8 ± 10.2 years) (Table 1). There was a significant inter-sex difference in mean ECD (male: 2762 ± 241 cells/mm^2^, female: 2719 ± 234 cells/mm^2^, *P* < 0.001), CV (male: 41.55 ± 6.70%, female: 43.29 ± 6.63%, *P* < 0.001), and HEX (male: 47.25 ± 7.20%, female: 44.77 ± 7.14%, *P* < 0.001). These inter-sex difference were similarly observed across the age groups except for participants with age ≥ 80 years (not shown in the tables). Excluding participants with history of contact lenses wear did not alter these numbers materially (not shown in the tables).

**Table 1:** Participant demographics.

Age range (years)	Male (n = 2331)	Female (n = 3382)	*P* value (male vs. female)	All participant (n = 5713)
40–49	275	496		771
50–59	319	652		971
60–69	958	1465		2423
70–79	680	701		1381
≥ 80	99	68		167
Age (years)^a^	64.2 ± 10.3	61.8 ± 10.2	< 0.001	62.8 ± 10.3
Height (cm)^a^	164.8 ± 6.5	152.5 ± 6.2	< 0.001	157.5 ± 8.7
Weight (kg)^a^	64.8 ± 9.9	53.0 ± 8.6	< 0.001	57.8 ± 10.8
Body mass index (kg/m^2^)^a^	23.8 ± 0.3	22.8 ± 0.3	< 0.001	23.2 ± 0.3
Antihypertensive drug use, n (%)	849 (36.4)	852 (25.2)	< 0.001	1701 (29.8)
Diabetes mellitus, n (%)	246 (10.6)	156 (4.6)	< 0.001	402 (7.0)
Dyslipidemia, n (%)	360 (15.4)	823 (24.3)	< 0.001	1183 (20.7)
Spherical equivalent (D)^a^	− 0.54 ± 2.34	− 0.63 ± 2.67	0.23	− 0.59 ± 2.54
Keratometric power (D)^a^	44.5 ± 1.5	43.8 ± 1.4	< 0.001	44.2 ± 1.5
IOP (mm Hg)^a^	13.5 ± 2.8	13.8 ± 2.7	< 0.001	13.7 ± 2.8

*D* diopter, *IOP* intraocular pressure, *n* number.

^a^Mean ± standard deviation.

### Characteristics of the participants stratified by SER

Age-adjusted sex-specific clinical characteristics according to SER was shown in Table 2. In male participants, age, height, keratometric power, IOP (intraocular pressure) and contact lenses wear showed statistically significant difference, whereas age, keratometric power, and contact lenses wear showed statistically significant difference in female participants.

**Table 2:** Age adjusted sex-specific participant characteristics according to spherical equivalent refraction.

SER (D)	Hyperopia	Emmetropia	Mild myopia	High myopia	P-value
	0.5≤	− 0.5≤ to < 0.5	− 6< to ≤ − 0.5	≤ − 6	
**Male**					
Age (years)^a^	69.0 ± 7.0	63.5 ± 10.1	60.4 ± 11.3	59.3 ± 11.2	< 0.001
Height (cm)	163.2 ± 0.2	163.7 ± 0.2	164.3 ± 0.3	166.1 ± 0.3	< 0.001
Weight (kg)	63.6 ± 0.4	63.8 ± 0.4	62.2 ± 0.4	66.5 ± 0.5	0.47
Body mass index (kg/m^2^)	23.8 ± 0.1	23.8 ± 0.1	22.9 ± 0.1	24.0 ± 0.2	0.11
Antihypertensive drug use, n (%)	357 (44.2)	224 (38.5)	243 (28.9)	25 (25.0)	0.056
Diabetes mellitus, n (%)	107 (13.3)	62 (10.7)	71 (8.4)	6 (6.0)	0.37
Dyslipidemia, n (%)	137 (17.0)	92 (15.8)	119 (14.1)	12 (12.0)	0.80
Keratometric power (D)	43.5 ± 0.07	43.9 ± 0.06	43.8 ± 0.06	43.9 ± 0.09	< 0.001
IOP (mmHg)	13.0 ± 0.1	13.0 ± 0.1	13.5 ± 0.2	15.2 ± 0.2	< 0.001
HbA1c (%)	5.8 ± 0.03	5.9 ± 0.03	5.8 ± 0.03	5.7 ± 0.03	0.33
Contact lenses wear, n (%)	2 (0.24)	2 (0.34)	49 (5.8)	21 (25.9)	< 0.001
**Female**					
Age (years)^a^	67.3 ± 7.0	61.2 ± 9.6	57.1 ± 10.5	55.1 ± 10.0	< 0.001
Height (cm)	150.5 ± 0.3	151.3 ± 0.2	151.5 ± 0.2	154.5 ± 0.3	0.76
Weight (kg)	51.2 ± 0.3	52.3 ± 0.3	52.3 ± 0.2	53.7 ± 0.6	0.078
Body mass index (kg/m^2^)	22.6 ± 0.1	22.7 ± 0.2	22.8 ± 0.1	22.5 ± 0.2	0.053
Antihypertensive drug use, n (%)	411 (33.2)	185 (23.9)	216 (18.5)	40 (20.1)	0.90
Diabetes mellitus, n (%)	73 (5.9)	41 (5.3)	32 (2.7)	10 (5.0)	0.18
Dyslipidemia, n (%)	383 (30.9)	196 (25.3)	209 (17.9)	35 (17.9)	0.40
Keratometric power (D)	44.3 ± 0.05	44.4 ± 0.06	44.7 ± 0.05	44.7 ± 0.1	< 0.001
IOP (mmHg)	13.5 ± 0.1	13.5 ± 0.1	13.5 ± 0.1	14.0 ± 0.2	0.76
HbA1c (%)	5.7 ± 0.02	5.7 ± 0.02	5.7 ± 0.02	5.7 ± 0.02	0.57
Contact lenses wear, n (%)	6 (0.47)	2 (0.26)	242 (20.7)	117 (70.5)	< 0.001

Age-adjusted mean ± standard error, or age-adjusted percentages presented unless otherwise indicated.

*D* diopter, *IOP* intraocular pressure, *n* number.

^a^Unadjusted mean ± standard deviation.

### Associations between the abnormal value of ECD, CV, HEX and SER

As shown in Table 3, there was no statistically significant relationships between SER and endothelial parameters in male participants. Whereas in female participants, compared to emmetropia, SER ≤ − 6 diopters (D) had significantly higher multivariable adjusted odds ratio (OR) of having the abnormal value of CV (OR = 2.07, 95% CI 1.39–3.10) and HEX (OR = 2.04, 95% CI 1.29–3.23). Further adjustment for contact lenses wear as a mediating factor partly attenuated these associations (Table 4). Of note, these association were not changed when excluding persons with contact lenses wear, although the associations were no longer statistically significant probably due to small number of cases in SER ≤ − 6 D (n = 49), with the respective multivariable adjusted OR of having the abnormal value of CV (OR = 1.51, 95% CI 0.75–3.01) and HEX (OR = 1.73, 95% CI 0.82–3.65) (not shown in the Tables). Association between SER and ECD was not statistically significant.

**Table 3:** Adjusted odds ratio of having the abnormal value of endothelial cell density, coefficient of variation, and hexagonality according to spherical equivalent refraction in male participants.

SER	Abnormal value of ECD			
	Abnormal/normal case (n)	OR^a^	OR^b^	OR^c^
0.5D≤	6/820	0.56 (0.18–1.78)	0.60 (0.19–1.90)	0.60 (0.19–1.92)
− 0.5D< to < 0.5D	6/576	1.0	1.0	1.0
− 6D< to ≤ − 0.5D	4/838	0.52 (0.14–1.85)	0.54 (0.15–1.95)	0.55 (0.15–1.99)
≤ − 6D	1/80	1.44 (0.17–12.23)	1.49 (0.17–13.05)	1.82 (0.21–16.14)
Total	17/2314			

SER	Abnormal value of CV			
	Abnormal/normal case (n)	OR	OR^b^	OR^c^
0.5D≤	421/405	0.89 (0.72–1.11)	0.85 (0.69–1.06)	0.85 (0.68–1.05)
− 0.5D< to < 0.5D	311/271	1.0	1.0	1.0
− 6D< to ≤ − 0.5D	471/371	1.12 (0.90–1.38)	1.15 (0.92–1.43)	1.11 (0.89–1.38)
≤ − 6D	41/40	0.91 (0.57–1.44)	0.95 (0.59–1.52)	0.77 (0.47–1.26)
Total	1244/1087			

SER	Abnormal value of HEX			
	Abnormal/normal case (n)	OR	OR^b^	OR^c^
0.5D≤	476/350	0.77 (0.62–0.97)	0.74 (0.59–0.93)	0.74 (0.59–0.93)
− 0.5D< to < 0.5D	370/212	1.0	1.0	1.0
− 6D< to ≤ − 0.5D	532/310	0.99 (0.79–1.23)	1.02 (0.81–1.28)	1.01 (0.81–1.27)
≤ − 6D	48/33	0.84 (0.52–1.35)	0.89 (0.55–1.45)	0.87 (0.53–1.44)
Total	1426/905			

*ECD* endothelial cell density, *CV* coefficient of variation, *HEX* hexagonality, *n* number, *D* diopter, *OR* odds ratio.

^a^Adjusted for age.

^b^Further adjusted for intraocular pressure, keratometric power, height, and antihypertensive drug use.

^c^Further adjusted for a history of contact lenses wear.

**Table 4:** Adjusted odds ratio of having the abnormal value of endothelial cell density, coefficient of variation, and hexagonality according to spherical equivalent refraction in female participants.

SER	Abnormal value of ECD			
	Abnormal/normal case (n)	OR^a^	OR^b^	OR^c^
0.5D≤	19/1254	2.86 (0.83–9.86)	2.88 (0.83–9.95)	2.94 (0.85–10.15)
− 0.5D< to < 0.5D	3/772	1.0	1.0	1.0
− 6D< to ≤ − 0.5D	6/1162	1.64 (0.41–6.62)	1.63 (0.40–6.60)	1.76 (0.43–7.14)
≤ − 6D	2/164	4.47 (0.73–23.5)	4.34 (0.70–27.1)	7.20 (0.96–54.01)
Total	30/3352			

SER	Abnormal value of CV			
	Abnormal/normal case (n)	OR^a^	OR^b^	OR^c^
0.5D≤	777/496	0.95 (0.79–1.15)	0.94 (0.78–1.14)	0.93 (0.77–1.13)
− 0.5D< to < 0.5D	489/286	1.0	1.0	1.0
− 6D< to ≤ − 0.5D	765/403	1.08 (0.89–1.31)	1.11 (0.91–1.34)	1.04 (0.85–1.27)
≤ − 6D	130/36	2.03 (1.36–3.03)	2.07 (1.39–3.10)	1.61 (1.03–2.51)
Total	2161/1221			

SER	Abnormal value of HEX			
	Abnormal/normal case (n)	OR^a^	OR^b^	OR^c^
0.5D≤	906/367	1.03 (0.84–1.27)	1.02 (0.83–1.26)	1.02 (0.83–1.25)
− 0.5D< to < 0.5D	560/215	1.0	1.0	1.0
− 6D< to ≤ − 0.5D	894/274	1.18 (0.96–1.46)	1.21 (0.98–1.51)	1.16 (0.93–1.44)
≤ − 6D	141/25	1.99 (1.26–3.14)	2.04 (1.29–3.23)	1.68 (0.96–2.78)
Total	2501/881			

*ECD* endothelial cell density, *CV* coefficient of variation, *HEX* hexagonality, *n* number, *D* diopter, *OR* odds ratio.

^a^Adjusted for age.

^b^Further adjusted for intraocular pressure, keratometric power, height, and antihypertensive drug use.

^c^Further adjusted for a history of contact lenses wear.

## Discussion

In this large population-based cross-sectional study, we found that in female participants, high myopia was associated with a higher prevalence of abnormal value of CV and HEX, compared to emmetropia, whereas no associations between high myopia and corneal endothelial abnormalities was shown in male participants. Association between SER and ECD was not clarified due to the minimal number of participants with abnormal value of ECD included in the analysis.

Mean ECD in normal ophthalmic participants were 2737 ± 238 cells/mm^2^ in this Japanese population. Previous population-based studies^8–19,33^ conducted in Western countries, India, Pakistan and Nigeria, mean ECD in participants over the age of 40 was 2300–2700 cells/mm^2^, and 1900–2000 cells/mm^2^ in Iran, while in East Asian countries (Japan, China, Singapore, and Philippine), it was 2700–3200 cells/mm^2^, which corresponded well to the present findings. These findings suggest that there are interracial differences in mean ECD. Our findings also showed the lower ECD in older participants, which was consistent with previous findings that ECD loss occurs with the normal aging process^8–23,27^. The present study also showed that mean ECD in male was greater than that of female when over demographic factors were not adjusted; however, the opposite result was shown in study conducted in Kumejima, Japan^16^. It remains controversial whether there is a relationship between sex and mean ECD^10,17–22^.

Our study also showed an inter-sex difference in CV (higher in female participants) and HEX (lower in female participants), which can be partially explained by the greater mean ECD in male participants. Few studies have reported this inter-sex difference in corneal endothelial morphology; however, one population study in Thailand showed that female participants had a higher CV than male participants, which is consistent with our results^21^. The abnormalities of endothelial parameters shown in female participants might be related to the susceptibility of the endothelial cells, and this can be a possible explanation of the relationships between high myopia and abnormal endothelial morphology shown in female participants in our study.

We found that female participants with high myopia had significantly higher OR of having the abnormal value of CV and HEX, compared to those with emmetropia, and the adjustment for contact lenses wear partly attenuated these associations. This suggested that the relationships between high myopia and abnormal value of CV and HEX may be mediated by, but not be fully explained by contact lenses wear, which is reported to affect corneal endothelial morphology^27^. Some studies reported that high myopia was an independent factor that affect ECD and corneal endothelial morphology. A previous study by Chang et al. showed a correlation between longer axial length and both low ECD and flatter corneal curvature in young participants with a mean age of 22.2 years without any history of contact lenses wear^25^. Another study, by Delshad et al., showed that mean ECD and HEX were lower in moderate myopia compared with low myopia in young participants with a mean age of 21.6 years without any history of contact lenses wear^26^. On the other hand, Sheng et al. reported the relationship between myopia and the abnormalities of both CV and HEX, without any relationship between myopia and ECD in participants 19–71 years of age, regardless of contact lenses wear^27^.

There might be some possible explanations about the relationships between myopia and the abnormalities of endothelial parameters. Myopia leads to the elongation of the eye ball^34^, which is driven largely by vitreous chamber depth increase^35^. On the other hand, it was reported that the longer axial length was related to flatter corneal curvature^25^. Since corneal endothelial cells lose mitotic activity postnatally^36^, it is speculated that the corneal endothelial cells flatten to cover the inner surface of the cornea in elongated eyes^25^, which may lead to reduced ECD^34^ temporarily during myopia progression in young population. In order to examine the influence of corneal curvature on endothelial morphology, we included keratometric power in the logistic regression models, and the correlations between high myopia and the abnormal value of CV and HEX were still statistically significant in female participants, regardless of keratometric power. Hence, we speculate that not only the elongation of the axial length and a concomitant changes in corneal curvature, but also other mechanisms may involve in the correlation between high myopia and the abnormal endothelial morphology seen in previous report^25–27^, and in female participants in our study. Recently, high myopia has been suggested to be an inflammation-related disease^37^, with one study reporting the proinflammatory status of high myopic cataract eyes^38^. It has been demonstrated that inflammatory cytokine elevations in aqueous humor was related to ECD loss^39^. Therefore, we speculate that high myopia may have a microenvironment that affects endothelial cells via intraocular cytokine changes. One study showed that galanin-immunoreactivity, a neuropeptide activity was prevalent in normal choroid and the retina^40^, and many neuropeptides are known to have anti-inflammatory actions^41^. Since choroids become thinner in more myopic eyes^42^, it is interesting to speculate that the changes in choroidal homeostasis in myopia might be related to the abnormalities of endothelial cells via intraocular neuropeptide changes.

The strength of the present study is that we included more than 5000 participants from the general population, which reflect high representativeness. So far, this is the largest epidemiological study in the world which examine this issue. However, there are some limitations in our study. First, it was a cross-sectional study, so we cannot determine the causal relationship. Second, we cannot deny the possibility that there might be some unmeasured confounding factors affecting the endothelial morphology. Third, in the questionnaire, we did not include outdoor activity which might affect not only ECD and endothelial morphology^16^, but also related to myopia progression^32^. Fourth, since we only included Japanese participants, generalizability to other race and ethnicity was uncertain.

In conclusion, we found that high myopia was associated with prevalence of abnormal value of CV and HEX in a community-based residents of Japanese female aged 40 years or older, while no relationships between myopia and the abnormal value of endothelial morphology was shown in male participants. Association between myopia and ECD was not clarified in this study.

## Methods

### Study population

This study was conducted as a population-based cross-sectional epidemiologic survey among Chikusei city residents as a part of the Japan Public Health Center-based Prospective Study for the Next Generation (JPHC-NEXT) Eye Study. Chikusei city is located in Ibaraki prefecture, Japan (eastern longitude of 139° 58′ and northern latitude of 36° 18′, approximately 70 km north of Tokyo), and has a population size of approximately 100,000.

All of the residents of Chikusei city whose ages were 40 years or more were eligible to participate in the annual health screening, and a total of 7098 participant who had taken ocular examination between 2013 and 2015 were included in this study. Information on current histories of antihypertensive drug use, diabetes and dyslipidemia were collected via face-to-face interview. Histories of ocular disease, surgery, and contact lenses wear were also obtained. The ocular examination included refractive status and intraocular pressure, using a combined refractometer and tonometer (Tonoref II, Nidek, Aichi, Japan). Central corneal thickness and corneal endothelium density were measured using noncontact specular microscope (Noncon Robo FA-3809D, Konan, Hyogo, Japan), and an average of multiple measurements were reported.

**Figure 1:**
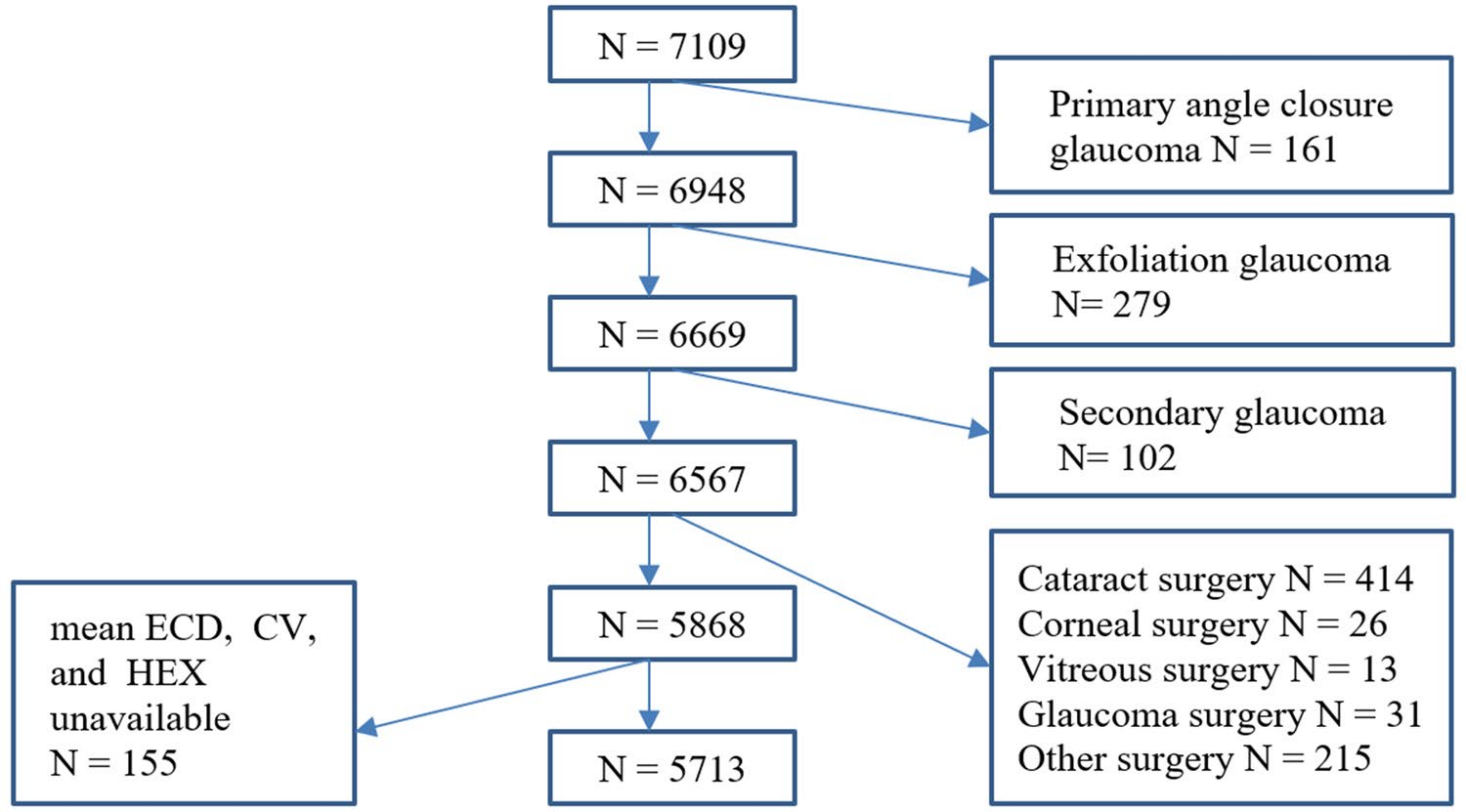
Flow chart of the study participants. Among 7109 participants, 542 eyes with histories of primary angle closure glaucoma, exfoliation glaucoma, and secondary glaucoma, and 699 eyes with ocular surgical histories were excluded. A total 155 eyes with unavailable mean endothelial cell density, coefficient of variation, and hexagonality data were also excluded from the analysis. *ECD* endothelial cell density, *CV* coefficient of variation, *HEX* hexagonality.

Of 7109 participants, 1241 participants with a prior history of primary angle closure glaucoma, exfoliation glaucoma, secondary glaucoma, and ocular surgeries, and 155 participants with uncountable or missing mean corneal ECD and morphology data were excluded. In the end, 5713 participants were included in the present study (Fig. 1). Of 5713 participants, 441 participants had a history of contact lenses wear, and most of them were myopes.

The JPHC-NEXT Eye Study followed the tenets of the Declaration of Helsinki, and the Institutional Review Boards of Osaka University, University of Tsukuba, National Cancer Center and Keio University approved the protocol. All participants provided written informed consent.

### Assessment of SER

The most commonly used threshold value for high myopia is a SER of ≤ − 6.00 D^43^, whereas the threshold of emmetropia still varies in different studies^44,45^. Since the SER of large number of population go between -0.5D and 0.5D in our study, we categorized the refraction into four categories: (1) emmetropia (> − 0.50 D to < 0.50 D), (2) slight to moderate myopia (> − 6.00 D to < − 0.50 D), (3) high myopia (≤ − 6.00 D), and (4) hyperopia (> 0.50 D).

### Assessment of corneal endothelial cell density and morphology

The density of endothelial cells per square millimeter from the central cornea was averaged across to calculate the total ECD. The variation of individual cell areas was calculated as CV, and the percentage of hexagonal cells was calculated as HEX.

ECD under 2000 cells/mm was considered as abnormal, since ECD does not decrease to less than 2000 cells/mm under the normal aging process^46^, and a donor cornea with an ECD less than 2000 cells/mm is not suitable for corneal transplant^46^. The abnormal value of CV was classified as CV not less than 40%, and the abnormal value of HEX was defined as HEX no more than 50%, because the risk of ECD loss after intraocular surgery is high in this range^46^.

### Statistical analysis

Inter-sex difference in major characteristics were assessed with an unpaired *t* test or a chi-square test. The overall difference in participant characteristics across the refraction categories was tested by analysis of covariance. The sex-specific association of SER with prevalence of abnormal value of ECD, CV, and HEX were determined using the logistic regression models adjusting for potential confounders (age, intraocular pressure, keratometric power, height, and antihypertensive drug use). As a potential mediator, we further adjusted for a history of contact lenses wear. All statistical tests were performed by Stata/SE 14.1 (StataCorp LLC, TX, USA), except for the evaluation of age-adjusted sex-specific clinical characteristics according to SER, which was conducted by SAS version 9.4 statistical software (SAS Institute Inc. Cary, NC, USA). The value of *P* < 0.05 was set as the threshold of significance.
